# *N*-(4-Methyl­phen­yl)succinimide

**DOI:** 10.1107/S1600536810001546

**Published:** 2010-01-16

**Authors:** B. S. Saraswathi, B. Thimme Gowda, Sabine Foro, Hartmut Fuess

**Affiliations:** a Department of Chemistry, Mangalore University, Mangalagangotri 574 199, Mangalore, India; b Institute of Materials Science, Darmstadt University of Technology, Petersenstrasse 23, D-64287 Darmstadt, Germany

## Abstract

In the mol­ecule of the title compound, C_11_H_11_NO_2_, the dihedral angle between the aromatic ring and the amide segment is 57.3 (1)°.

## Related literature

For a related structure, see: Saraswathi *et al.* (2010 ▶).
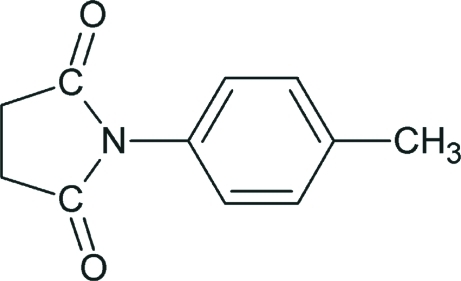


## Experimental

### 

#### Crystal data

C_11_H_11_NO_2_
*M* *_r_* = 189.21
Monoclinic, 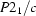

*a* = 13.543 (3) Å
*b* = 5.6539 (9) Å
*c* = 13.365 (3) Å
β = 109.35 (2)°
*V* = 965.6 (3) Å^3^
*Z* = 4
Mo *K*α radiation
μ = 0.09 mm^−1^
*T* = 299 K
0.44 × 0.24 × 0.08 mm

#### Data collection

Oxford Diffraction Xcalibur diffractometer with a Sapphire CCD detector
Absorption correction: multi-scan (*CrysAlis RED*; Oxford Diffraction, 2009 ▶) *T*_min_ = 0.961, *T*_max_ = 0.993
3389 measured reflections
1924 independent reflections
1262 reflections with *I* > 2σ(*I*)
*R*_int_ = 0.020

#### Refinement

*R*[*F* ^2^ > 2σ(*F* ^2^)] = 0.068
*wR*(*F* ^2^) = 0.136
*S* = 1.23
1924 reflections
160 parameters
Only H-atom coordinates refined
Δρ_max_ = 0.17 e Å^−3^
Δρ_min_ = −0.16 e Å^−3^

### 

Data collection: *CrysAlis CCD* (Oxford Diffraction, 2009 ▶); cell refinement: *CrysAlis RED* (Oxford Diffraction, 2009 ▶); data reduction: *CrysAlis RED*; program(s) used to solve structure: *SHELXS97* (Sheldrick, 2008 ▶); program(s) used to refine structure: *SHELXL97* (Sheldrick, 2008 ▶); molecular graphics: *PLATON* (Spek, 2009 ▶); software used to prepare material for publication: *SHELXL97*.

## Supplementary Material

Crystal structure: contains datablocks I, global. DOI: 10.1107/S1600536810001546/ng2719sup1.cif

Structure factors: contains datablocks I. DOI: 10.1107/S1600536810001546/ng2719Isup2.hkl

Additional supplementary materials:  crystallographic information; 3D view; checkCIF report

## Figures and Tables

**Fig. 1.**
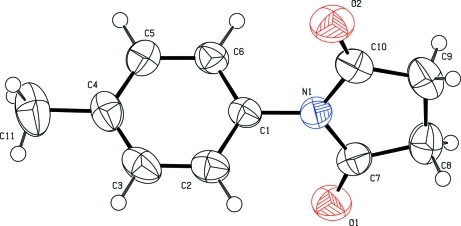
Molecular structure of the title compound, showing the atom labelling scheme. The displacement ellipsoids are drawn at the 50% probability level. The H atoms are represented as small spheres of arbitrary radii.

**Fig. 2.**
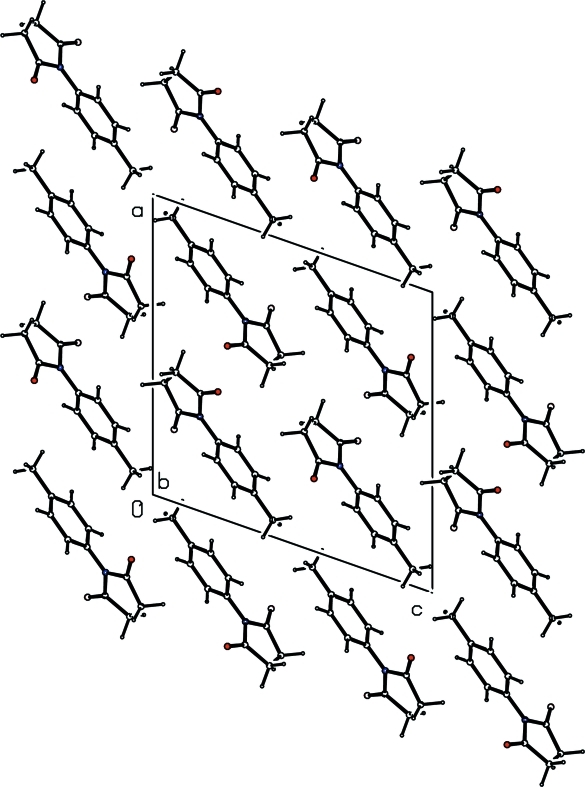
Molecular packing of the title compound.

## supplementary crystallographic information

### Comment

As a part of studying the effect of ring and side chain substitutions on the crystal structures of amides (Saraswathi *et al.*, 2010), the crystal structure of *N*,*N*-(4-methylphenyl)succinimide has been determined (I) The molecule lies nearly on a twofold rotation axis that passes through the N and C*_para_* atoms as well as through the mid-point of the methylene C atoms. The dihedral angle between the benzene ring and the amide segment in the molecule is 57.3 (1)° (Fig. 1)..

The torsional angles of the groups, C2 - C1 - N1 - C7, C2 - C1 - N1 - C10, C6 - C1 - N1 - C7 and C6 - C1 - N1 - C10 in the molecule are 59.0 (3)°, -121.8 (3)°, -120.2 (3)° and 59.0 (3)°, respectively.

The packing of molecules into row like infinite chains in the *ac*-plane is shown in Fig.2.

### Experimental

The solution of succinic anhydride (0.02 mole) in toluene (25 ml) was treated dropwise with the solution of 4-methylaniline (0.02 mole) also in toluene (20 ml) with constant stirring. The resulting mixture was stirred for one hour and set aside for an additional hour at room temperature for the completion of reaction. The mixture was then treated with dilute hydrochloric acid to remove the unreacted 4-methylaniline. The resultant solid *N*-(4-methylphenyl)succinamic acid was filtered under suction and washed thoroughly with water to remove the unreacted succinic anhydride and succinic acid. It was recrystallized to constant melting point from ethanol. *N*-(4-Methylphenyl)succinamic acid was then heated for 2 h and then allowed to cool slowly to room temperature to get crystals of *N*-(4-methylphenyl)succinimide. The purity of the compound was checked and characterized by its infrared spectra. The plate like colourless single crystals of the compound used in X-ray diffraction studies were grown in ethanolic solution by slow evaporation at room temperature.

### Refinement

The H atoms were located in difference map, and their positional parameters were refined freely [C—H = 0.92 (4)–0.99 (3) Å].

### Crystal data

**Table:**

C_11_H_11_NO_2_	*F*(000) = 400
*M**_r_* = 189.21	*D*_x_ = 1.302 Mg m^−^^3^
Monoclinic, *P*2_1_/*c*	Mo *K*α radiation, λ = 0.71073 Å
Hall symbol: -P 2ybc	Cell parameters from 1028 reflections
*a* = 13.543 (3) Å	θ = 2.6–27.8°
*b* = 5.6539 (9) Å	µ = 0.09 mm^−^^1^
*c* = 13.365 (3) Å	*T* = 299 K
β = 109.35 (2)°	Plate, colourless
*V* = 965.6 (3) Å^3^	0.44 × 0.24 × 0.08 mm
*Z* = 4	

### Data collection

**Table:**

Oxford Diffraction Xcalibur diffractometer with a Sapphire CCD detector	1924 independent reflections
Radiation source: fine-focus sealed tube	1262 reflections with *I* > 2σ(*I*)
graphite	*R*_int_ = 0.020
Rotation method data acquisition using ω and φ scans.	θ_max_ = 26.4°, θ_min_ = 3.1°
Absorption correction: multi-scan (*CrysAlis RED*; Oxford Diffraction, 2009)	*h* = −16→11
*T*_min_ = 0.961, *T*_max_ = 0.993	*k* = −7→7
3389 measured reflections	*l* = −16→15

### Refinement

**Table:**

Refinement on *F*^2^	Primary atom site location: structure-invariant direct methods
Least-squares matrix: full	Secondary atom site location: difference Fourier map
*R*[*F*^2^ > 2σ(*F*^2^)] = 0.068	Hydrogen site location: difference Fourier map
*wR*(*F*^2^) = 0.136	Only H-atom coordinates refined
*S* = 1.23	*w* = 1/[σ^2^(*F*_o_^2^) + (0.0241*P*)^2^ + 0.6535*P*] where *P* = (*F*_o_^2^ + 2*F*_c_^2^)/3
1924 reflections	(Δ/σ)_max_ = 0.017
160 parameters	Δρ_max_ = 0.17 e Å^−^^3^
0 restraints	Δρ_min_ = −0.16 e Å^−^^3^

### Special details

**Table:**

Experimental. CrysAlis RED (Oxford Diffraction, 2009) Empirical absorption correction using spherical harmonics, implemented in SCALE3 ABSPACK scaling algorithm.
Geometry. All e.s.d.'s (except the e.s.d. in the dihedral angle between two l.s. planes) are estimated using the full covariance matrix. The cell e.s.d.'s are taken into account individually in the estimation of e.s.d.'s in distances, angles and torsion angles; correlations between e.s.d.'s in cell parameters are only used when they are defined by crystal symmetry. An approximate (isotropic) treatment of cell e.s.d.'s is used for estimating e.s.d.'s involving l.s. planes.
Refinement. Refinement of *F*^2^ against ALL reflections. The weighted *R*-factor *wR* and goodness of fit *S* are based on *F*^2^, conventional *R*-factors *R* are based on *F*, with *F* set to zero for negative *F*^2^. The threshold expression of *F*^2^ > σ(*F*^2^) is used only for calculating *R*-factors(gt) *etc*. and is not relevant to the choice of reflections for refinement. *R*-factors based on *F*^2^ are statistically about twice as large as those based on *F*, and *R*- factors based on ALL data will be even larger.

### Fractional atomic coordinates and isotropic or equivalent isotropic displacement parameters (Å^2^)

**Table:**

	*x*	*y*	*z*	*U*_iso_*/*U*_eq_	
C1	0.25083 (19)	0.2010 (5)	0.2351 (2)	0.0412 (6)	
C2	0.2495 (2)	0.0189 (5)	0.3031 (2)	0.0531 (8)	
H2	0.293 (2)	−0.118 (5)	0.306 (2)	0.064*	
C3	0.1863 (3)	0.0374 (6)	0.3657 (2)	0.0602 (9)	
H3	0.187 (2)	−0.089 (5)	0.412 (2)	0.072*	
C4	0.1243 (2)	0.2332 (6)	0.3618 (2)	0.0547 (8)	
C5	0.1272 (2)	0.4126 (6)	0.2926 (3)	0.0556 (8)	
H5	0.086 (2)	0.550 (5)	0.290 (2)	0.067*	
C6	0.1896 (2)	0.3988 (5)	0.2296 (2)	0.0486 (7)	
H6	0.193 (2)	0.523 (5)	0.182 (2)	0.058*	
C7	0.3049 (2)	0.0071 (5)	0.0936 (2)	0.0484 (7)	
C8	0.3859 (3)	0.0509 (7)	0.0415 (3)	0.0609 (9)	
H8A	0.440 (2)	−0.074 (6)	0.068 (2)	0.073*	
H8B	0.353 (2)	0.041 (6)	−0.037 (3)	0.073*	
C9	0.4333 (3)	0.2887 (7)	0.0825 (3)	0.0631 (9)	
H9A	0.510 (2)	0.288 (5)	0.109 (2)	0.076*	
H9B	0.411 (2)	0.408 (6)	0.032 (2)	0.076*	
C10	0.3921 (2)	0.3502 (5)	0.1706 (2)	0.0512 (7)	
C11	0.0552 (3)	0.2544 (9)	0.4292 (3)	0.0859 (14)	
H11A	−0.013 (3)	0.301 (7)	0.394 (3)	0.103*	
H11B	0.085 (3)	0.350 (7)	0.492 (3)	0.103*	
H11C	0.047 (3)	0.100 (7)	0.452 (3)	0.103*	
N1	0.31460 (16)	0.1858 (4)	0.16839 (17)	0.0437 (6)	
O1	0.24145 (17)	−0.1496 (4)	0.07658 (16)	0.0637 (6)	
O2	0.41793 (16)	0.5111 (4)	0.23272 (19)	0.0714 (7)	

### Atomic displacement parameters (Å^2^)

**Table:**

	*U*^11^	*U*^22^	*U*^33^	*U*^12^	*U*^13^	*U*^23^
C1	0.0456 (15)	0.0390 (15)	0.0363 (14)	−0.0054 (12)	0.0099 (12)	−0.0001 (12)
C2	0.0603 (19)	0.0461 (17)	0.0485 (17)	−0.0008 (15)	0.0121 (15)	0.0052 (15)
C3	0.076 (2)	0.059 (2)	0.0431 (17)	−0.0155 (18)	0.0166 (16)	0.0073 (16)
C4	0.0559 (17)	0.069 (2)	0.0411 (16)	−0.0183 (16)	0.0181 (14)	−0.0093 (16)
C5	0.0612 (19)	0.0545 (19)	0.0546 (18)	0.0017 (15)	0.0235 (16)	−0.0047 (16)
C6	0.0588 (18)	0.0438 (16)	0.0435 (17)	0.0016 (14)	0.0175 (14)	0.0057 (14)
C7	0.0562 (17)	0.0465 (16)	0.0396 (15)	0.0043 (15)	0.0119 (13)	−0.0003 (14)
C8	0.061 (2)	0.075 (2)	0.0497 (19)	0.0061 (17)	0.0223 (16)	0.0011 (18)
C9	0.0533 (18)	0.079 (3)	0.058 (2)	−0.0011 (18)	0.0192 (16)	0.0168 (19)
C10	0.0413 (15)	0.0518 (18)	0.0542 (18)	−0.0005 (14)	0.0071 (13)	0.0077 (16)
C11	0.081 (3)	0.123 (4)	0.063 (2)	−0.031 (3)	0.038 (2)	−0.019 (3)
N1	0.0451 (12)	0.0428 (13)	0.0436 (13)	−0.0032 (11)	0.0152 (10)	−0.0036 (11)
O1	0.0827 (15)	0.0526 (13)	0.0565 (13)	−0.0154 (12)	0.0239 (11)	−0.0110 (11)
O2	0.0679 (14)	0.0578 (14)	0.0843 (16)	−0.0163 (12)	0.0194 (12)	−0.0155 (13)

### Geometric parameters (Å, °)

**Table:**

C1—C2	1.378 (4)	C7—N1	1.397 (3)
C1—C6	1.380 (4)	C7—C8	1.502 (4)
C1—N1	1.434 (3)	C8—C9	1.511 (5)
C2—C3	1.385 (4)	C8—H8A	0.99 (3)
C2—H2	0.96 (3)	C8—H8B	0.99 (3)
C3—C4	1.380 (4)	C9—C10	1.502 (4)
C3—H3	0.94 (3)	C9—H9A	0.99 (3)
C4—C5	1.381 (4)	C9—H9B	0.94 (3)
C4—C11	1.504 (5)	C10—O2	1.203 (3)
C5—C6	1.380 (4)	C10—N1	1.394 (3)
C5—H5	0.95 (3)	C11—H11A	0.92 (4)
C6—H6	0.95 (3)	C11—H11B	0.97 (4)
C7—O1	1.202 (3)	C11—H11C	0.95 (4)
			
C2—C1—C6	120.1 (3)	C9—C8—H8A	109.3 (18)
C2—C1—N1	120.5 (3)	C7—C8—H8B	109.8 (17)
C6—C1—N1	119.3 (2)	C9—C8—H8B	114.8 (19)
C1—C2—C3	119.2 (3)	H8A—C8—H8B	111 (3)
C1—C2—H2	119.0 (17)	C10—C9—C8	105.5 (3)
C3—C2—H2	121.7 (17)	C10—C9—H9A	110.0 (18)
C4—C3—C2	121.8 (3)	C8—C9—H9A	113.6 (19)
C4—C3—H3	120.0 (18)	C10—C9—H9B	106.7 (19)
C2—C3—H3	118.2 (19)	C8—C9—H9B	112.5 (19)
C3—C4—C5	117.6 (3)	H9A—C9—H9B	108 (3)
C3—C4—C11	122.2 (3)	O2—C10—N1	124.3 (3)
C5—C4—C11	120.3 (3)	O2—C10—C9	128.1 (3)
C6—C5—C4	121.7 (3)	N1—C10—C9	107.5 (3)
C6—C5—H5	119.5 (19)	C4—C11—H11A	116 (2)
C4—C5—H5	118.7 (18)	C4—C11—H11B	114 (2)
C1—C6—C5	119.5 (3)	H11A—C11—H11B	110 (3)
C1—C6—H6	118.4 (16)	C4—C11—H11C	106 (2)
C5—C6—H6	122.1 (17)	H11A—C11—H11C	103 (3)
O1—C7—N1	124.1 (3)	H11B—C11—H11C	107 (3)
O1—C7—C8	128.3 (3)	C10—N1—C7	112.9 (2)
N1—C7—C8	107.6 (3)	C10—N1—C1	123.4 (2)
C7—C8—C9	105.5 (3)	C7—N1—C1	123.7 (2)
C7—C8—H8A	106.5 (18)		
			
C6—C1—C2—C3	−0.1 (4)	C8—C9—C10—N1	−9.4 (3)
N1—C1—C2—C3	−179.3 (3)	O2—C10—N1—C7	−175.6 (3)
C1—C2—C3—C4	0.1 (5)	C9—C10—N1—C7	5.1 (3)
C2—C3—C4—C5	−0.1 (5)	O2—C10—N1—C1	5.1 (4)
C2—C3—C4—C11	179.8 (3)	C9—C10—N1—C1	−174.2 (2)
C3—C4—C5—C6	−0.1 (5)	O1—C7—N1—C10	−178.2 (3)
C11—C4—C5—C6	−180.0 (3)	C8—C7—N1—C10	1.5 (3)
C2—C1—C6—C5	0.0 (4)	O1—C7—N1—C1	1.1 (4)
N1—C1—C6—C5	179.2 (3)	C8—C7—N1—C1	−179.2 (2)
C4—C5—C6—C1	0.1 (5)	C2—C1—N1—C10	−121.8 (3)
O1—C7—C8—C9	172.3 (3)	C6—C1—N1—C10	59.0 (3)
N1—C7—C8—C9	−7.4 (3)	C2—C1—N1—C7	59.0 (3)
C7—C8—C9—C10	10.1 (3)	C6—C1—N1—C7	−120.2 (3)
C8—C9—C10—O2	171.3 (3)		
